# The great pretender: Multi-system tuberculosis and pathological fracture masquerading as a severe acute football groin injury – Case study with a 5-year follow-up

**DOI:** 10.17159/2078-516X/2023/v35i1a13980

**Published:** 2023-06-02

**Authors:** M Lichaba, W Diesel, D Constantinou

**Affiliations:** 1 Department of Exercise Science and Sports Medicine (DESSM), School of Therapeutic Sciences, Faculty of Health Sciences, University of Witwatersrand, South Africa; 2 International Federation of Sports Medicine Collaborating Centre of Sports Medicine, Johannesburg, South Africa

**Keywords:** extrapulmonary tuberculosis, HIV, AIDS, acetabular fracture

## Abstract

In this clinical case, a man presented with a groin injury on his dominant side, which he apparently sustained in football (soccer) practice on the previous day. The man was unable to walk unassisted and had to be transported in a wheelchair. The consulting practitioner grew suspicious upon finding minimal clinical evidence and nothing notable on the X-ray to suggest a severe acute injury. A subsequent detailed workup revealed extrapulmonary tuberculosis (EPTB) of the musculoskeletal (MSK) and genitourinary tract (GUT) systems, complicated by a pathological fracture of the acetabulum, as the cause of the groin injury. Management of the EPTB resolved the condition with no relapse nor long-term sequelae beyond five years, despite being immunocompromised. We present the clinical case and a five year follow-up. The case serves as a reminder of the possibility that other conditions may mimic sports injuries and further illustrates a rare presentation of such a condition.

Groin injuries account for 2 – 5% of all sports injuries and up to 8% among footballers.^[1]^. Ice hockey and football present the highest risk for groin injuries, (10 – 11% of all injuries in these sports are groin injuries). Men seem more susceptible to groin injuries than women. The differential diagnosis for groin pain includes intra-abdominal pathology, genitourinary abnormalities, referred pain from the lumbo-sacral spine and hip pathology.^[2]^ The coexistence of two or more of these conditions is frequent, with unclear diagnosis in 30% of cases. ^[2]^ In cases of hip pathology, acetabular fractures are an extremely rare aetiology, especially amongst footballers. Acetabular fractures usually happen in high-velocity impact, associated dislocation, adolescent players, and/or osteoporotic/osteopenic bone ^[3]^. Rankin, Bleakly and Cullen^[4]^ in a 6-year review reported hip pathology to be the leading cause of groin pain in the sporting population. However, in the list of hip pathology, labral tears feature at 33%, (second to femoral-acetabular impingement), and no mention at all of acetabular fractures. Though acetabular fractures are rare in soccer, the hip is among the joints that tuberculosis (TB) commonly affects. There are reports and warnings of non-sports injuries presenting as sports injuries, including sacroiliac TB, oncological conditions, and rheumatological conditions.

Tuberculosis (TB) is a very old infectious disease with anthropological evidence of the mycobacterium seen since the 9^th^ millennium. It is still a significant disease, being among the top 10 causes of death worldwide,^[5]^ and remains the leading cause of death in the world from a single infectious disease, ranking higher than HIV/AIDS.^[5]^ TB’s highest disease burden is in adult men (20 – 39 years of age), accounting for more than half the cases.^[5]^

Tuberculosis had been declining in the last century, but there has been an increase in recent times due to the advent of the HIV pandemic, the destruction of socio-cultural support systems resulting from urbanization, and worsening conditions of our public health systems and programs. Tuberculosis is the most common illness among people living with HIV, who are 16 – 27 times more likely to acquire TB.^[5]^ It is also the leading cause of death amongst these people, accounting for a third of all HIV-related deaths. Africa ranks second in the world, with 24% of the total global TB cases.^[5]^ Sub-Saharan Africa has the highest number of people living with HIV/AIDS, with South Africa being amongst the top 10 countries in the world.

Kwan and Ernst^[6]^ refer to HIV and TB as the ‘deadly human syndemic”. They found in their review that the likelihood of getting extrapulmonary tuberculosis (EPTB) increases with immune compromise, particularly when CD4 counts fall below 350 cells/mm^3^. They quote the bone and GUT as the highest sites affected by EPTB.

TB is often difficult to diagnose in patients with very low CD4 counts as the body’s immune response is compromised and traditional pathology tests sometimes fail to detect TB. EPTB is even harder to diagnose as it commonly presents with symptoms and signs related to the affected organs and systems, which then mimic afflictions typically affecting such organs and systems. TB is generally a more insidious low-grade infection, even more so in EPTB, especially when infected individuals have a compromised immune system. Hence, the diagnosis of EPTB may require more extensive and invasive investigations

We present the first known reported case of EPTB presenting as a football groin injury.

## Case report

A 40-year-old male consulted his sports physician one day after sustaining a right groin injury when he slipped and fell into a split position while playing football. The patient was immediately not able to be ambulated due to the severe groin pain. He required assistance and was transported in a wheelchair. The patient did not recall hearing a ‘snap’ or a ‘click’ at the time of injury. He also did not collide with anyone. On examination, the patient was in severe distress due to pain. He was of average athletic build with no signs of cachexia or muscle atrophy. He was apyrexial, not anaemic, not clubbed and pink. The peripheral pulses were all normal. There were no skin changes or deformities observed. No shotty nor matted lymph nodes were palpable on examination, either generally or in the inguinal area. The right groin area was extremely tender to palpation in the area of the anterior hip joint, but no masses nor defects were palpable. There was no “real” leg length discrepancy. He was extremely tense on examination of the right leg, so the reflexes were hard to assess. Sensation was normal. The patient was very reluctant to actively move the right hip at all. The log roll test reproduced the patient’s groin pain, worse on internal rotation, but it did not produce any sound. The Thomas’ test was limited to 30 degrees of hip flexion, while the FABER test reproduced the groin pain and significantly limited external rotation but no sacro-iliac joint (SIJ) discomfort. The FADIR caused groin tenderness on internal rotation. The Compression test also reproduced severe groin pain. The Straight Leg Raise test was negative. The knee joint examination was unremarkable. General systemic examination did not reveal any abnormalities other than a small (<1cm diameter) ganglion on the dorsum of the left wrist. He also complained of a recent painless left scrotal swelling, confirmed clinically as a soft but firm mass.

The patient’s past medical history confirmed he was HIV positive on antiretroviral therapy (ARVs), and revealed poorly controlled hypertension with Stage 3 Chronic Renal Failure (CRF), (Table 1). Monitoring done six months before the injury had shown a CD4 count of 276 cells/mm^3^ and an undetected viral load (VL) of less than 40 cps/ml. On presentation, the CD4 count had fallen to 137, with an undetectable VL (Table 1).

### Special investigations

**Fig. 1:**
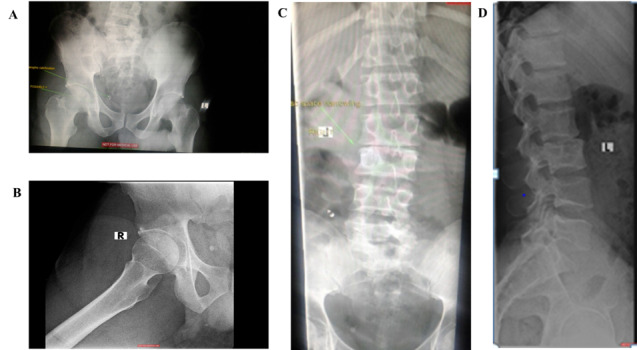
(A) X-ray of the pelvis AP view; (B) X-ray of the right hip AP view; (C) Lumbar spine X-ray, AP view; (D) Lumbar spine X-ray lateral view

Ultrasound imaging of the affected groin revealed no significant injury to the soft tissues or muscles (Figure 1A). Plain X-rays of the right hip (Figure 1B) revealed dystrophic calcification in the right pelvis and a lucency through the acetabulum. X-ray of the lumbar spine showed disc space narrowing with disc degeneration across L2/L3 (Figures 1C and D).

**Fig. 2:**
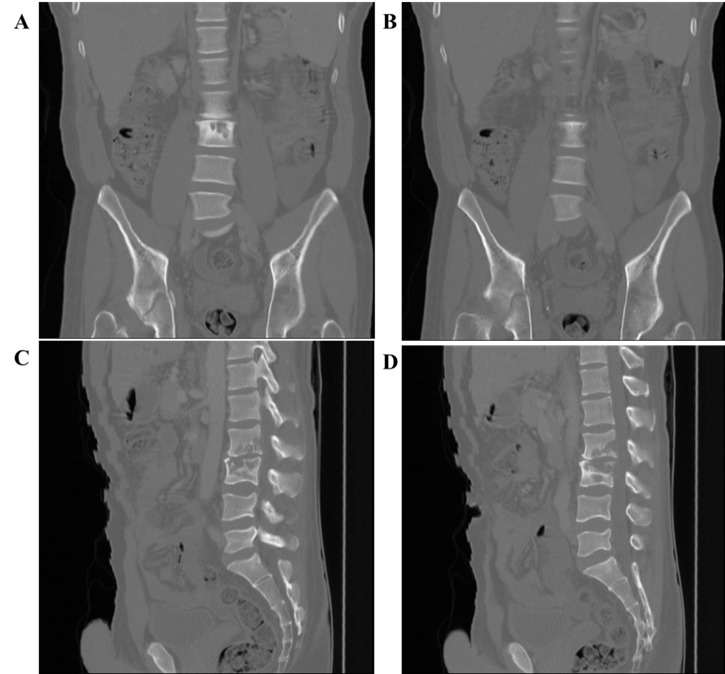
CT scan lumbar spine and pelvis AP and lateral views

A CT scan of the abdomen and pelvis (Figure 2) delayed due to financial constraints and performed a month later, revealed osteolytic lesions of L2/L3 (Figures 2A – D) and the right acetabulum, (Figures 2A and B) with pathological fractures and associated calcification of the right acetabulum (Figures 2A and B).

**Fig. 3:**
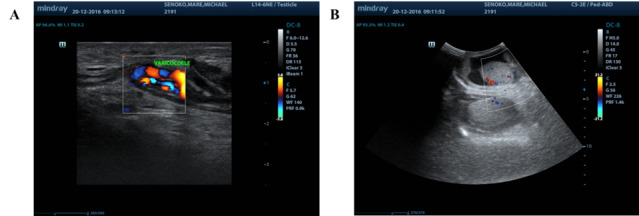
Ultrasound of the left scrotum

An ultrasound of the scrotum (Figure 3) showed a left acute epididymal-orchitis with an associated large left hydrocele and varicoceles.

### Case management

The patient was managed with nonsteroidal anti-inflammatories whilst being investigated. A month after the initial consultation, the patient returned complaining that the wrist ganglion became significantly bigger (>2cm diameter) and requested it be removed. During the gangliectomy the surgeon observed rice bodies. Histology and synovium culture confirmed mycobacterium tuberculosis.

A hydrocoelectomy was performed with the urologist finding solid caseous material typical of TB necrosis, in the left epididymis, with TB confirmed histologically. The definitive diagnosis of TB MSK and GUT was then made in support of multi-organ EPTB which included the spine and the hip, consistent with the radiological features.

The patient was referred to the local municipal clinic for anti-TB therapy. At 9 months, the clinic sister erroneously stopped his treatment and on realising this the doctor re-commenced the treatment and concluded the recommended 12 months course for EPTB. He continued with his ARV and antihypertensive treatment. The orthopaedic complications responded well to conservative treatment with analgesia and physiotherapy, requiring no surgery.

### Outcome/follow up

At 12 months post-treatment initiation, the patient had recovered fully and was deemed cured of TB. All the orthopaedic complications, including the acetabular fracture, had healed clinically. No control radiology was deemed necessary

The inflammatory markers CRP and ESR also normalised. The CD4 count improved from 137 to 330 cells/mm^3^ (Table 1). Unfortunately, the blood pressure control remained poor, and the renal function deteriorated warranting dialysis (Table 1). He survived a coma secondary to hypertensive encephalopathy and pulmonary oedema 3 years after his groin pain presentation. At 5 years follow-up, his VL remained undetected, the CD^4^ count had risen above 350/cmm^3^ and the inflammatory markers were below 10 units (Table 1). He returned to work full-time with no clinical sequelae of the multisystem TB or encephalopathy. He continued haemodialysis three times a week for CRF. He did not resume playing football or engaging in any physical activity due to his fear of injury. He began to consider himself an “invalid” because of the frequent need for dialysis. He was advised to engage in a chronic disease rehabilitation program appropriate for his clinical and metabolic status, which he unfortunately never took up.

### Main points to consider

Though hip pathology is a common cause of groin pain, acetabular fractures feature least in the list of hip aetiologies for sports-related groin disruptions. Acetabular fractures are typically caused by high-velocity trauma or low bone strength. Although acetabular fractures are very rare in soccer, there are well-documented cases in the literature of them occurring during recreational games even at low velocity with no contact. Most of these injuries happen with the position of risk being hip flexion in internal rotation, resulting in posterior fracture-dislocations, with total functional failure acutely ^[5]^. In our case, the TB probably caused osteolysis of the bone thus making the acetabulum vulnerable to break from a relatively low velocity, indirect injury.

Tuberculosis and HIV are highly prevalent in South Africa, which means that practitioners in any speciality must be vigilant for HIV and TB-related complications, even in young and active individuals. Our patient fits the profile of being at high risk for such complications, given his demographic and geographic profile.

While it is important to consider sporting participation in active young individuals, a high index of suspicion should be maintained if the clinical picture does not fit the expected presentation, if the symptoms are exaggerated, or if there is an unsatisfactory response to conventional treatment. Moreover, this patient would have likely benefited from continuing to participate in sports. Unfortunately, he did not pursue it due to fear of reinjury, medical complications, and loss of physical prowess.

## Conclusion

This case reminds us of the insidious, atypical nature of the presentation of TB, especially in immunocompromised individuals. It also highlights how immunocompromising systemic or local afflictions may predispose to otherwise very rare conditions such as acetabular fractures, causing major tissue damage and disability from relatively low-velocity force. Sports physicians should be cognisant of the role of infections in the aetiology of health disruptions of athletes, especially in the African (Sub – Saharan) context. The focus on aetiology should not be limited to trauma alone. A broad-minded, holistic approach to athletes' challenges, even if presenting as musculoskeletal disturbances, is critical. A comprehensive systemic review of presenting symptoms, signs, and general medical history is mandatory. Acetabular fractures though rare in sports cause total dysfunction and can result in severe long-term sequelae and should not be missed.

## Figures and Tables

**Table 1:** Summary of clinical and pathology results

Clinical pathology parameter	6 months before injury	At time of presentation	6 months post-treatment	9 months post-treatment*	On resumption of treatment	At completion of treatment	28 months later - admission for coma	8 months post-coma	5 year final follow up
**BP (mmHg)**	160/100 ˠ	170/100 ˠ	130/100 ˠ	NM	NM	160/110 ˠ	140/85	165/100 ˠ	177/104
**CRP (mg/l)**	NA	86.1 ˠ	NM	10.5	23.6 ˠ	9.3	181.2 ˠ	NM	7.6
**ESR (mm/h)**	NA	74 ˠ	NM	44 ˠ	35 ˠ	21 ˠ	NM	NM	9.0
**eGFR (ml/min/1.73m** **^2^** **)**	47 ˠ	36 ˠ	48 ˠ	40 ˠ	NM	31 ˠ	4 ˠ	4 ˠ	3 ˠ
**VL (cps/ml)**	< 40	< 40	< 40	NM	NM	< 40	< 40	< 40	20
**CD4 cell count (cells/mm** **^3^** **)**	276 ˠ	137 ˠ	332 ˠ	NM	NM	330 ˠ	380 ˠ	458 ˠ	380 ˠ

* indicates TB treatment erroneously stopped.

ˠ indicates data out of reference range.

BP, blood pressure; CRP, C-reactive protein; ESR, erythrocyte sedimentation rate; eGFR, estimated glomerular filtration rate; VL, HIV Viral load; CD4, CD4 lymphocyte count; NA, not applicable, NM, not measured.
